# Surgical Management of Gastric Gastrointestinal Stromal Tumours: Comparison of Outcomes for Local and Radical Resection

**DOI:** 10.1155/2018/2140253

**Published:** 2018-06-21

**Authors:** Anantha Madhavan, Alexander W. Phillips, Claire L. Donohoe, Rebecca J. Willows, Arul Immanuel, Mark Verril, S. Michael Griffin

**Affiliations:** ^1^Northern Oesophagogastric Cancer Unit, Royal Victoria Infirmary, Newcastle upon Tyne NE1 4LP, UK; ^2^Northern Centre for Cancer Care, Freeman Hospital, Newcastle upon Tyne NE7 7DN, UK

## Abstract

Gastrointestinal stromal tumours (GISTs) most commonly originate from the stomach. Their treatment is dependent on size and whether they are symptomatic. Curative treatment requires surgery, which may be preceded by neoadjuvant imatinib if it is felt that this will aid in achieving clear (R0) resection margins. The aim of this study was to evaluate outcomes from patients that underwent a “local” organ-preserving operation, with those that required a more radical resection, and the influences on selecting a more radical resection. A retrospective review of patients undergoing surgery for symptomatic gastric GISTs from a single institution over 9 years was carried out. Patients were divided into three cohorts dependent on whether they had a “local” resection, “anatomical” resection, or “extended” resection. 71 patients were included. Overall, 5-year survival was 92%. Operating time, blood loss, and length of stay were significantly lower in the group undergoing local resection (*p* < 0.05). Tumour size was also smaller in the local group (median 4 cm versus 5 cm *p* < 0.05). Tumour location also influenced the type of surgery performed, with tumours at the cardia, gastroesophageal junction, and antrum all having “anatomical” resections. Lymphadenectomy did not appear to impact on outcomes. These findings indicate that local excision, where possible, does not impair oncological outcomes.

## 1. Introduction

Gastrointestinal stromal tumours (GISTS) account for 1% of gastrointestinal malignancies [1]. They are mesenchymal in origin with the majority (60–70%) occurring in the stomach [2]. Presentation and management of gastric GISTs is dependent on the size, location, and presentation. Small GISTs, up to 2 cm in size, may be found incidentally and are frequently asymptomatic. They usually do not require surgical intervention, but rather surveillance to ensure they are not significantly enlarging [3]. Larger GISTs (greater than 5 cm) and those that are causing either obstructive symptoms or have bled require excision [4, 5].

The curative treatment of gastric GISTs involves surgical resection [3, 6, 7]. The aim of surgery is to achieve an R0 resection (no evidence of microscopic residual disease and negative margins) with an intact pseudocapsule [8]. The size of the tumour, location, and its proximity to adjacent intra-abdominal viscera may influence the surgical approach undertaken and the extent of stomach resected [7–10]. Whilst a localised surgical resection may be employed for some tumours, it may be preferable to perform a more conventionally “radical” approach including a lymphadenectomy for others. Current guidelines state that lymphadenectomy is “normally not required” but do not unequivocally state indications for lymphadenectomy [11]. In some instances, en bloc resection of adjacent organs may be required to obtain a clear resection margin [3]. In the last decade, imatinib has been used in the neoadjuvant setting to reduce the size of the primary tumour in order to improve the success of obtaining a clear resection margin [12, 13]. Laparoscopic surgery is increasingly used to facilitate excision of small or medium sized GISTs below 5 cm in size; however, this can be challenging, and care is required when handling the tumour to try and minimise the risk of rupture which may compromise oncological outcomes [14, 15].

The aim of this study was to evaluate outcomes of patients requiring surgery for gastric GISTs from a single institution. Three cohorts that underwent either a “local” excision, anatomical resection, or an extended resection were compared, and factors influencing the extent of surgery were reviewed; role of lymphadenectomy and suitability of a local resection for those who received neoadjuvant imatinib were evaluated.

## 2. Method

### 2.1. Patient Population

A retrospective review of all patients undergoing curative surgery for a gastric GIST was performed. Patients were treated at a single centre (Northern Oesophagogastric Unit, Newcastle upon Tyne, UK) between January 2007 and January 2016. All patients underwent standard staging investigations which consisted of endoscopic assessment, endoscopic ultrasound, and CT, were then discussed in a multidisciplinary team meeting (MDM), and subsequently underwent either local resection (either open or laparoscopically), anatomical resection usually in the form of a total or subtotal gastrectomy, (with lymphadenectomy), or an extended anatomical or extended local resection (en bloc resection of all involved structures and lymphadenectomy). The principles of selection of a local operative approach included the ability to obtain an R0 resection margin and preservation of function where possible. In tumour encroaching on the cardia or the incisura or pylorus, a formal anatomical resection was performed (subtotal or total gastrectomy with Roux-en-Y reconstruction with lymphadenectomy or proximal partial gastrectomy with jejunal interposition reconstruction). Anatomical resection included at least a D1 lymphadenectomy as routine in those patients undergoing either a total or subtotal resection. Where tumours invaded surrounding structures, a resection was considered if R0 resection was possible with en bloc removal of invaded structures (distal pancreas, spleen, diaphragm, or colon) and classified as an extended resection.

**Figure 1:**
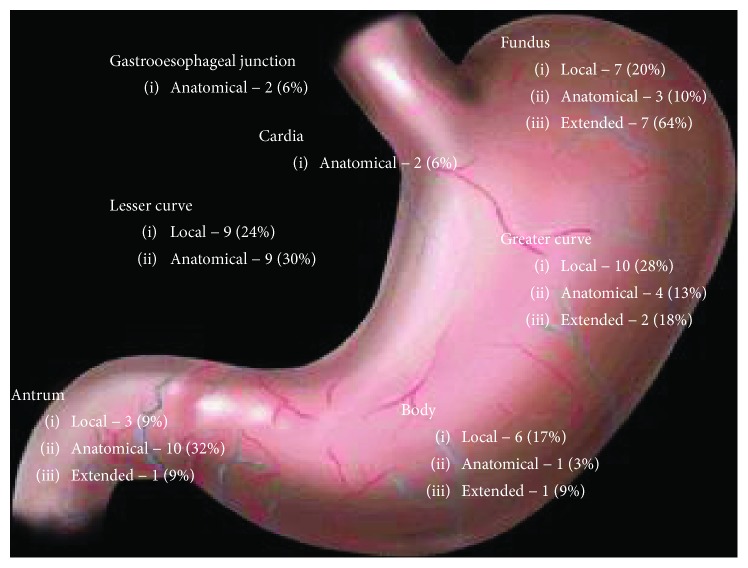
Distribution of gastrointestinal tumours in the anatomical, local, and extended resection cohort.

Where a tumour was deemed not resectable without unacceptable morbidity, treatment with neoadjuvant imatinib was used at a starting dose of 400 mg/day. Treatment response was assessed by CT scan using the Choi criteria every 3 months [16]. The decision to proceed to surgery was when the tumour became resectable or if the tumour did not respond to the standard neoadjuvant treatment regimen. The algorithm for surgical decision-making is summarised in Figure 1.

### 2.2. Analysis

To assess the impact of operative approach on outcome, patients were divided into three cohorts: local excision, anatomical resection, and extended en bloc resection.

For analysis of impact of lymphadenectomy on outcome, patients were divided into three cohorts for analysis: those undergoing a resection where the underlying aim was to excise the tumour only, with adequate margins and no purposeful lymphadenectomy were regarded as “local excision”; and those where a conventional oncological resection was carried out were regarded as an “anatomical resection”. This cohort included both patients who underwent a subtotal and total gastrectomy with either a D1 or D2 lymphadenectomy and patients who underwent a proximal partial gastrectomy with no formal lymphadenectomy. The third group was the extended resection cohort. In this cohort, extension of the tumours into adjacent abdominal viscera required an en bloc resection of the organ to achieve a clear resection margin. This was either combined with a local or anatomical resection.

Data were prospectively recorded using a standardised pro forma. Complications were assessed for twice daily and categorised using the Accordion classification system [17]. Pathological stage groups were assessed using the Miettinen and Lasota classification system [18].

### 2.3. Statistical Analysis

Statistical calculations were performed by SPSS software, version 22 (SPSS, Chicago, IL).

A Kruskal-Wallis test and Mann–Whitney *U* test were used to compare continuous variables, and categorical data were compared using a chi-squared/Fisher's exact test. A log-rank (Mantel-Cox) test was used to compare survival between groups. Survival statistics were calculated using Kaplan-Meier method, and the log-rank test was used to assess differences in survival between groups. Overall, survival rate was recorded for patients as well as time to recurrence for disease-free survival. Survival time was measured from the date of operation to the date of an event or last follow-up. All tests performed were two-tailed. *p* values less than 0.05 (2-sided) were considered statistically significant.

## 3. Results

From January 2007 to January 2016, 76 patients underwent surgery for gastric GISTs. Operative mortality was 0%, and significant morbidity (classified as Accordion III or IV) was 5% with an overall recorded operative morbidity of 10.5%. The overall survival rates in the series at two and five years were 95% and 92%, respectively. Disease-free survival at two and five years was 95% and 92%, respectively.

### 3.1. Outcomes Based on the Extent of Surgical Resection

Patients were divided into three groups: local resection, anatomical resection, and extended resection. Surgeries carried out in each group are summarised in Table 1.

The baseline demographics (ASA, BMI, age, and gender) of those operated on were comparable between the groups, although those undergoing extended resection were more likely to be males (Table 2). The overall size of the tumour was smaller in the local excision cohort versus the anatomical and extended resection cohorts (*p* = <0.05).

Analysis of operative parameters revealed that there was significantly lower median operating time (100 minutes versus 180 minutes *p* < 0.05), blood loss (80 ml versus 255 ml *p* < 0.05), and length of stay (6 days versus 11 days *p* < 0.05) in the local excision cohort.

Most patients (70%) undergoing a local excision had their surgery performed laparoscopically. There was no significant difference in overall postoperative morbidity between the three cohorts. However, three (8.8%) patients in the local resection cohort had significant morbidity, classified as Accordion ≥ 3 compared to only one (3.3%) in the anatomical excision cohort. Pathological prognostic groups did not significantly differ between the groups (Table 2). R0 resection status was 97% in the local excision cohort and 97% in the anatomical resection group compared to 90% in the extended radical excision cohort (*p* = 0.572).

### 3.2. Impact of Location and Size on Surgery Performed

There was variation in the location of the tumours within the three cohorts. Tumours located within the cardia 2 (6%), gastrooesophageal junction 2 (6%), and antrum 10 (32%) underwent a formal anatomical resection. The choice of resection for tumours located within the lesser curve and fundus was influenced by the size.

For tumours located on the lesser curve, size appeared to influence whether a local resection or anatomical resection was performed. For those undergoing a local resection, median size was 4 cm (4–10 cm) versus 5 cm (5–15 cm) for those that underwent an anatomical resection (*p* = 0.22).

The median size of the tumour in the fundus in the local resection cohort was 4 cm (4–9 cm) versus 9 cm (8–10 cm) in the anatomical resection cohort (*p* = 0.02). There was a trend towards local resection for smaller tumours on the greater curve. The median tumour size in the local resection cohort was 4 cm (4-5 cm) versus 5 cm (5–10 cm) in the anatomical resection cohort but was not statistically significant (*p* = 0.12).

Extended resection was required to achieve a clear resection margin in tumours located in the fundus 7 (64%) and greater curve 2 (18%) due to extraluminal extension into adjacent viscera.

### 3.3. Role of Lymphadenectomy

In the anatomical and extended resection cohort, 30 patients had lymph nodes excised as part of their surgery. In the extended resection cohort, 9 patients (82%) had a lymphadenectomy with a median lymph node yield of 10 (6–48). In the anatomical resection cohort, 21 patients (70%) had lymphadenectomy with a median lymph node yield of 17 (4–54).

Only one patient had any positive nodes found at pathologic analysis. In this case, there was a single positive node out of twenty-four resected. This patient had undergone neoadjuvant imatinib for 12 months for a 10 cm fundal GIST invading the spleen and subsequently underwent a total gastrectomy with distal pancreatectomy and splenectomy. R0 resection margin was achieved, and the final pathology was low risk. After 12 months of adjuvant imatinib and follow-up of 24 months postoperatively, no disease recurrence was noted on surveillance CT.

### 3.4. Choice of Surgical Resection after Neoadjuvant Imatinib

Thirteen patients had neoadjuvant imatinib due the large size of the tumour and its close proximity to adjacent abdominal viscera, threatening the surgical resection margin. One patient underwent local excision, three patients underwent anatomical resection, and nine patients underwent extended resection due to extraluminal extension of the tumour into adjacent organs. The median duration of neoadjuvant treatment with imatinib was 12 months (range: 3–36 months) with a median percentage decrease in maximum diameter of −31.4% (range +10% to −67.3%).

Postoperative pathological risk of recurrence classification was found to be very low risk in 2, low in 5, intermediate in 1, and high in 5 patients. Two of the patients with low-risk tumours by size had no tumour cells at pathological analysis.

One patient had a palliative resection, as a peritoneal deposit was noted at the time of surgery and the resection margin was positive due to widespread extension of the primary tumour into adjacent organs. This patient died from metastatic disease at 11 months postoperatively despite treatment with adjuvant imatinib. Of the patients receiving neoadjuvant imatinib, six subsequently received adjuvant imatinib. Five out of the six patients had high-risk pathology, and one patient had intermediate risk.

Three patients developed recurrence in this cohort despite R0 resection, the median time to recurrence was 28 months (14–42). Two of the patients had high-risk pathology and had adjuvant imatinib. The remaining patient had low-risk pathology and thus did not receive adjuvant imatinib. All the patients developed solitary metastases in the liver and underwent metastasectomy.

The overall five-year survival was 91%, and the 5-year disease-free survival rate was 77% in patients who underwent neoadjuvant imatinib and surgery.

### 3.5. Prognosis and Survival

Six patients (8%) had metastatic recurrence of the GISTs during the follow-up period. One patient in the local excision group following an R0 resection with low-risk pathology developed peritoneal and liver metastases 47 months after surgery. The patient subsequently died of metastatic disease despite adjuvant treatment with imatinib.

Of the remaining five patients, three underwent an anatomical resection and two underwent an extended resection. Three of the patients had postoperative high-risk pathology, of which two received adjuvant treatment. The other two patients had intermediate-risk and low-risk pathology, respectively. The median time to recurrence was 21 months (4–40). Four of these patients had concurrent solitary liver metastases and underwent metastasectomy. The remaining patient developed solitary metastases in the sacrum five months after surgery and was treated with adjuvant imatinib.

**Figure 2:**
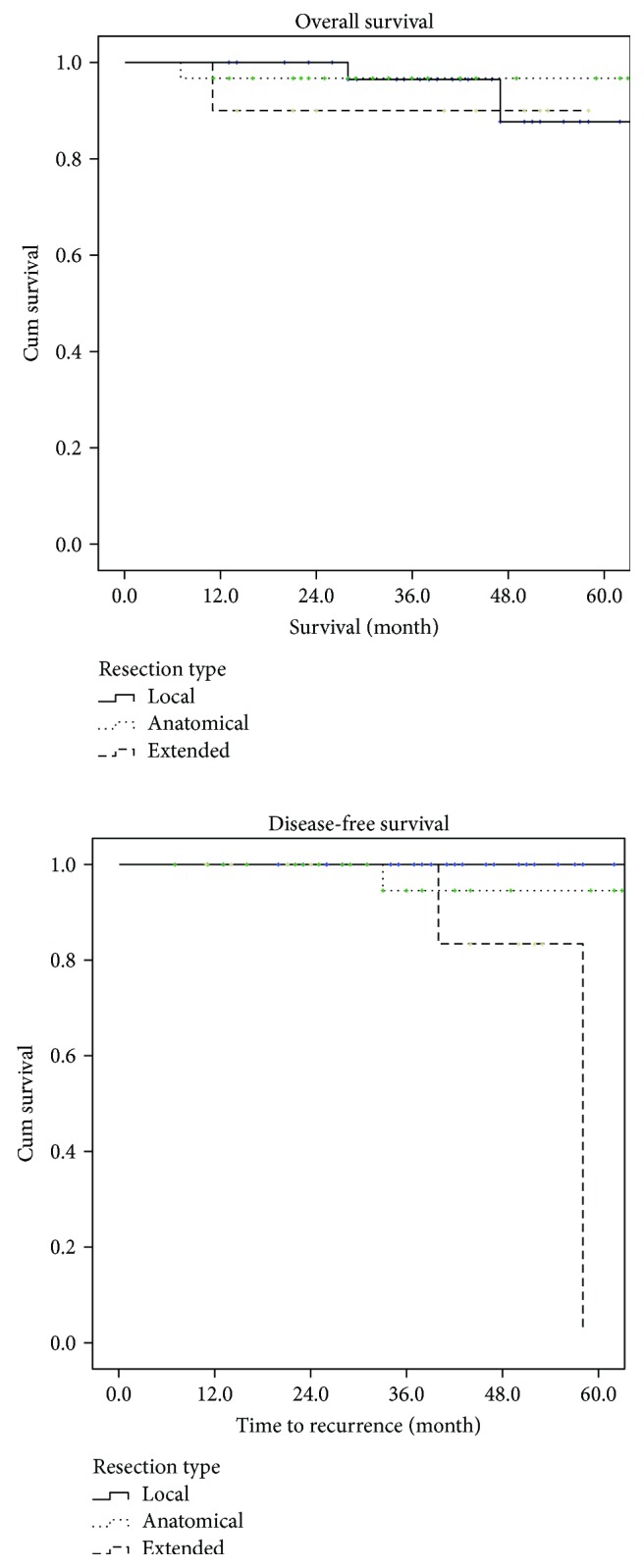
Overall survival and disease-free survival according to the surgery type.

**Figure 3:**
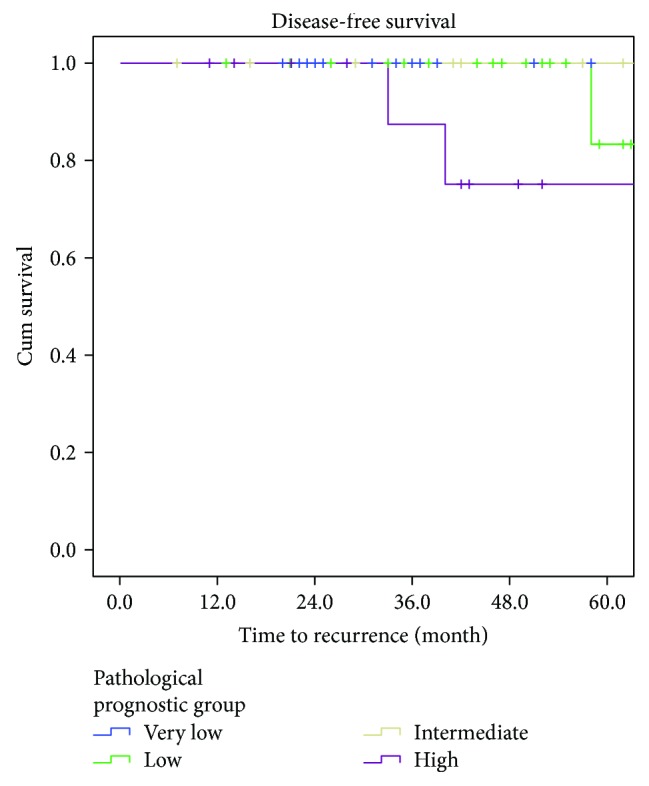
Survival according to the pathological prognostic group [18].

Only one patient had an incomplete resection (R1) in the local excision cohort. Postoperative pathology was low risk, and the patient remained tumour free over a 24-month surveillance period to date. Although tumour size and location influence the surgical approach taken in order to achieve an R0 resection status, none of the patients with R1 resection were found to have recurrence during surveillance. Overall survival did not significantly differ according to the operative approach with 5-year survival rates of 90%, 97%, and 90%, respectively (*p* = 0.716). The disease-free survival rate was lower in patients undergoing anatomical (57%) or extended resection (45%) compared to local resection (100%) (*p* = 0.012) (Figure 2). Recurrence-free survival is closely correlated with the pathological prognostic group, although there was one patient with low-risk pathology with disease recurrence at 56 months postoperatively (Figure 3).

## 4. Discussion

The findings from this study demonstrate that patient outcomes are not compromised by performing a local resection provided that an R0 resection can be achieved. In this series, all patients were discussed within an MDM to ensure agreement that a local excision, where deemed surgically feasible, was also oncologically appropriate. In this study, those undergoing a local excision are more likely to have the procedure performed laparoscopically and have significantly less blood loss, a shorter operating time, and a shorter stay in hospital.

**Figure 4:**
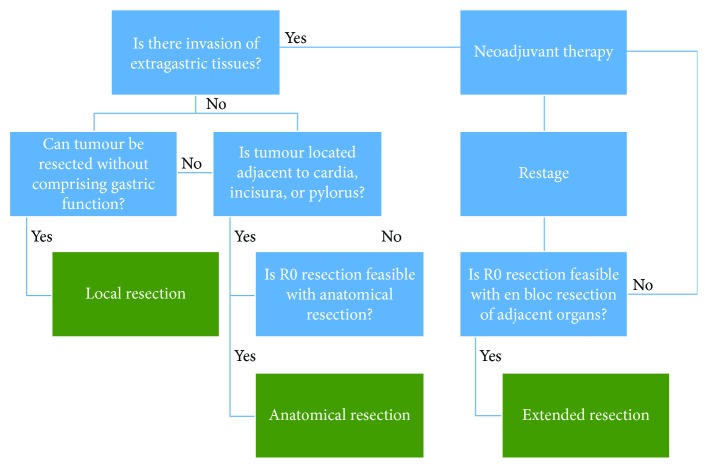
Algorithm for surgical decision-making for gastric GISTs.

Within this study, tumour location appeared to have the most important impact on influencing whether or not local excision was performed. Tumours located in the cardia and gastrooesophageal junction required a formal anatomical resection to maintain the gastrointestinal function. For tumours located in the lesser curve and antrum, an additional consideration for the choice of resection was the size of the tumour. Larger tumours located in these regions required a formal anatomical resection compared to a local excision to achieve both an R0 resection and to provide an acceptable functional outcome. The majority of tumours (65%) located in the fundus, greater curve, and body were amenable to local excision. An extended resection was required if there was evidence of extraluminal extension into adjacent abdominal viscera to achieve a clear resection margin. However, the findings from this study demonstrate that curative resection can be achieved in patients with large extragastric GISTs with associated invasion of surrounding abdominal viscera provided that clear margins can be obtained. For those tumours confined to the gastric wall, without local invasion, systematic lymphadenectomy is not regarded as necessary as gastric GISTs rarely metastasise to lymph nodes [19]. We have developed an algorithm for surgical decision-making for gastric GISTs based on our experience (Figure 4). As demonstrated in this cohort, only one patient had a positive node following a total gastrectomy and D2 lymphadenectomy. It is thus difficult to determine whether lymphadenectomy has a prognostic influence in patients where nodal involvement may exists. However, this extremely low positive nodal yield indicates that radical lymphadenectomy is likely to be rarely important.

Laparoscopic gastric GIST resection was first described more than a quarter of a century ago [20]. Previous studies have shown that laparoscopic resection is associated with less postoperative pain, less morbidity, and shorter hospital stay [10, 21]. These results are corroborated by the findings from the present study. However, concerns over the appropriateness of laparoscopic resection exist with tumours greater than 5 cm in size. The most recent NCCN guidelines suggest that larger tumours may be resected laparoscopically or laparoscopically assisted with a hand port dependent on size and location of the tumour [8]. The findings from this study demonstrate that a local excision can be achieved with larger tumours. However, it should be noted that there was no significant increase in morbidity in resections that included a conventional oncological lymphadenectomy.

Ensuring that the whole tumour, where possible, is resected and that tumour rupture is avoided is of paramount importance in patients requiring gastric GIST resection. In this study, those undergoing a radical approach had larger tumours and as would be expected were associated with a more aggressive pathology. Despite this, five-year survival was comparable between cohorts; however, disease-free survival was lower at 76% in the radical cohort compared to that at 90% in the local group. This may reflect that a more radical operation provides good disease control in those with more aggressive tumours.

This series supports the work by Rutkowski et al., which highlights the role of neoadjuvant imatinib in downstaging patients with large gastrointestinal tumours. In this study, 92% R0 resection was achieved in patients undergoing neoadjuvant imatinib followed by surgery. The median tumour size was 11 cm (6–22). Three patients developed isolated liver metastases and underwent metastasectomy. Despite this, the overall five-year survival in the R0 resection cohort was 100%. The study is limited because of the focus primarily on patients who underwent surgery following neoadjuvant imatinib treatment. The outcomes of the patients who did not proceed to surgery following neoadjuvant imatinib were not evaluated. This was due to the progression of the disease during neoadjuvant treatment, which meant a curative R0 resection was not surgically achievable. Thus, response to neoadjuvant imatinib could be a prognostic factor in large gastrointestinal tumours of the stomach. However, this hypothesis requires further evaluation.

In conclusion, local resection, preserving the stomach, provides excellent outcomes for patients requiring excision of a GIST. In those patients with potentially more aggressive tumours, radical resection is not associated with significantly increased morbidity and may provide good long-term disease control. Further, prospective trials are required to fully establish any benefit from more radical resection.

## Figures and Tables

**Table 1:** Surgical procedures.

Procedure	Number
*Local resections*	
Open sleeve gastrectomy	3
Lap sleeve gastrectomy	2
Open wedge resection	13
Laparoscopic wedge resection	15
*Anatomical resections*	
Subtotal gastrectomy	16
Subtotal gastrectomy + D2 lymphadenectomy	4
Total gastrectomy	3
Proximal partial gastrectomy	2
Middle third gastrectomy	2
Oesophagogastrectomy	1
Proximal gastrectomy + jejunal interposition	2
*Extended resections*	
Open sleeve gastrectomy + distal pancreatectomy + splenectomy	2
Open sleeve gastrectomy + wedge resection diaphragm	1
Total gastrectomy + en bloc resection of diaphragm	1
Total gastrectomy + distal pancreatectomy + splenectomy	4
Partial gastrectomy + splenectomy	1
Wedge resection + splenectomy	1
Subtotal gastrectomy + transverse colon resection	1

**Table 2:** Demographics.

	Local resection (*n* = 35)	Anatomical resection (*n* = 30)	Extended resection (*n* = 11)	*p* value
Age	64 (43–86)	68 (47–81)	58 (35–76)	0.03
BMI	28 (21–40)	25 (21–35)	26.5 (22–36)	0.403
Sex (M : F)	20 : 15	14 : 17	9 : 1	0.045
ASA				
1	3 (8.8%)	5 (16.7%)	1 (10%)	0.614
2	25 (73.5%)	19 (63.3%)	7 (64%)	
3	6 (17.6%)	5 (16.7%)	2 (18%)	
4	0	1 (3.3%)	1 (10%)	
Neoadjuvant treatment	1 (3%)	5 (16%)	7 (70%)	<0.001
Laparoscopic approach	19 (54.3%)	0	0	<0.001
Operating time (min)	90 (60–220)	180 (120–320)	170 (110–300)	<0.001
Blood loss (ml)	60 (50–80)	250 (50–1000)	550 (50–3800)	0.084
Length of stay (days)	6 (2–20)	10 (5–33)	10 (4–16)	<0.001
Postoperative complication	4 (15%)	2 (6.7%)	2 (18.2%)	0.465
Accordion > 3	3 (8.6%)	1 (3.3%)	0	0.34
Pathological size (cm)	3.5 (1.2–10.6)	5.0 (2.5–14.8)	7.0 (3.5–12.0)	0.021
Pathological risk group				
Very low	15 (42.9%)	8 (26.7%)	2 (18.2%)	0.235
Low	11 (31.4%)	10 (33.3%)	5 (45.5%)	
Medium	6 (17.1%)	5 (15.2%)	0	
High	3 (8.6%)	7 (23.3%)	4 (36.4%)	
Adjuvant treatment	1 (3%)	3 (10%)	3 (27%)	0.134
Lymph node yield	0 (0–19)	17 (0–54)	10 (0–48)	<0.001
Positive LN	0	1 (0-1)	0	
Resection margin				0.572
R0	34 (97.1%)	29 (96.7%)	10 (90.9%)	
R1	1 (2.9%)	1 (3.3%)	1 (9.1%)	
